# Acute effects of high load vs. low load combined with blood flow restriction on explosive performance and muscle-tendon unit mechanical properties

**DOI:** 10.1371/journal.pone.0353131

**Published:** 2026-07-07

**Authors:** Junjie Zhang

**Affiliations:** School of Sports Science and Physical Education, Nanjing Normal University, Nanjing, Jiangsu, China; Federation University Australia, AUSTRALIA

## Abstract

This study examined the acute effects of high-load resistance training and low-load resistance training with blood flow restriction on sprint ability, pennation angle and lower limb muscle stiffness. A randomized crossover trial was conducted in 17 collegiate male sprinters. Four intervention conditions were applied: high-load (90% 1RM hip thrust [HT] or half-squat [HS]) and low-load (30% 1RM HT or HS with blood flow restriction [BFR]) protocols. Muscle–tendon stiffness, pennation angle, and 20-m sprint performance were assessed at 5 minutes post-intervention. The results showed that statistically significant differences were observed among interventions (p < 0.05). Regarding sprint performance, the 30% HT + BFR significantly improved 0-20m sprint time, whereas the 30% HS + BFR significantly improved 10-20m sprint time compared to high-load conditions. In terms of muscle architecture, both 30% HS + BFR and 90% HS conditions elicited a significant decrease in the pennation angle of the rectus femoris. Moreover, muscle-tendon stiffness generally increased following conditioning activities; notably, the 30% HT + BFR condition increased rectus femoris stiffness, while the 30% HS + BFR condition significantly increased stiffness in the gastrocnemius and Achilles tendon compared to other interventions or baseline. In conclusion, this study demonstrates that low-load resistance exercise combined with blood flow restriction produces greater acute improvements in sprint performance in male sprinters than high-load training, accompanied by reductions in pennation angle and increases in muscle-tendon stiffness.

## Introduction

In competitive sports, especially sprint, athletes’ performance is highly dependent on the quality of their pre-race warm-ups. Post-activation performance enhancement (PAPE) refers to an acute improvement in voluntary muscular performance following a conditioning activity involving high-intensity voluntary contractions [1,2]. This enhancement is fundamentally driven by the contractile history of the muscle and is influenced by factors such as contraction intensity, volume, and the balance between resultant fatigue and potentiation mechanisms (e.g., muscle temperature, fluid shifts, and neural drive). Furthermore, previous research has demonstrated that conditioning contractions can induce acute changes in pennation angle [3,4]. Regarding the changes in pennation angle, Mahlfeld et al. (2004) [5] conducted ultrasonographic examinations on the lateral femoral muscle and observed changes in the pennation angle in three sets of examinations. The study investigated the effect of PAPE on pennation angle using ultrasonography and observed that although the change was not statistically significant, the pennation angle decreased by 0.5° immediately after three sets of 3 s of static maximal voluntary contraction (MVC) (15.7° vs. 16.2°). After 3–6 minutes of MVC practice, the pennation angle further decreased by 1.8° (14.4° vs. 16.2°). Theoretically, an increased pennation angle can reduce the efficiency of force transmission from muscle fibers to the tendon due to the angular orientation [6]. Therefore, the acute decrease in pennation angle observed in PAPE may enhance performance by allowing a greater proportion of the force generated by the fibers to be transmitted directly to the tendon, thereby improving mechanical efficiency. Additionally, Earp et al. (2011) [7] found that a larger pennation angle was associated with better early rate of force development during drop jumps, whereas Fukunaga et al. (1997) [8]and Mahlfeld et al. (2004) [5] reported that a smaller pennation angle enhances force transmission efficiency. These seemingly contradictory findings highlight the ongoing controversy regarding the relationship between pennation angle changes and athletic performance, underscoring the need for randomized controlled trials to resolve this issue.

A novel perspective suggests that acute enhancements in muscle-tendon stiffness may play a critical role in PAPE [9]. In addition to its role in rate of force development (RFD) and the storage and release of elastic energy, increased lower-limb muscle-tendon stiffness also plays an important role in force production. Previous studies indicate that higher stiffness facilitates the stretch-shortening cycle (SSC) and force transmission [10,11], as evidenced by the significant correlation observed between tendon stiffness and vertical jump performance [12]. While stiffness assessment tools (e.g., myotonometry) have been validated [13] and applied in athletic populations such as soccer and basketball players [14,15], the specific contribution of individual muscle-tendon stiffness to performance remains complex [16]. Papla et al. [17]showed that the stiffness of the Achilles tendon in athletes was significantly improved after barbell squat and deep jump exercise intervention, but Krzysztofik et al. [18]found that the stiffness of the vastus lateralis and Achilles tendon was even reduced within 2–10 minutes after acute high-intensity squat intervention.

While high-load resistance exercises are traditionally required, Davids et al. compared low-load blood flow restriction and high-load resistance exercise in trained individuals and found that low-load blood flow restriction is an effective alternative to high-load resistance exercise for obtaining muscle fitness in trained populations [19]. The substantial fatigue associated with such loads can counteract the potentiation effect [20]. This limitation necessitates the exploration of alternative protocols like BFR.

Blood flow restriction (BFR) has garnered significant attention from sports researchers, coaches, and athletes in recent years. For instance, Jarosz et al. (2023) found that applying blood flow restriction (BFR) to the rectus femoris muscle at rest significantly altered its mechanical properties, including stiffness and frequency [3]. BFR involves applying pressure during training to inhibit venous blood flow and partially restrict arterial blood flow, leading to inadequate oxygen transport in localized areas. This disruption causes a rapid increase in blood lactate levels and free radicals [21],which enhances the recruitment of high-threshold motor units [22]. The physiological mechanism underlying post-activation potentiation involves the phosphorylation of myosin light chains. The amount of tension generated during muscle contraction depends primarily on the number of activated cross-bridges and the increased recruitment of high-threshold motor units [23,24]. This acute increase in motor unit activation suggests that low-load blood flow restriction (BFR) exercise could induce a potentiation effect similar to that of high-load exercise, despite the lower mechanical load. This offers an alternative for athletes unable to engage in high-load resistance exercise during rehabilitation. However, it should be noted that although several studies and recent meta-analyses have examined the acute effects of low-load resistance exercise combined with BFR on performance outcomes, existing evidence remains heterogeneous and is largely derived from non-sprint-specific tasks or isolated performance measures [25,26]. Moreover, direct comparisons between low-load BFR exercise and traditional high-load resistance exercise, as well as concurrent assessments of muscle architecture and muscle-tendon stiffness, remain limited, particularly in trained sprinters. Therefore, this study aimed to comprehensively examine the acute effects of low-load resistance exercise combined with BFR versus high-load resistance exercise, performed as either hip thrusts or half squats, on sprint ability, pennation angle, and lower extremity muscle-tendon stiffness.

## Materials and methods

### Participants

An a priori sample size calculation was conducted using G*Power 3.1 software (Universities, Düsseldorf, Germany). The calculation was based on an F-test (ANOVA: Repeated measures, within factors) to align with the study’s crossover design. Based on previous research regarding the acute effects of PAPE on sprint performance [27], the effect size, significance level, and power were set at 0.40 [1,27], 0.05, and 0.80 [28], respectively. The effect size employed originates from comparable multi-condition crossover design studies, and key assumptions such as repeated-measures correlation and nonsphericity were considered in the estimation [1,29]. The analysis indicated that a minimum of 12 participants was required to detect a significant effect. Considering a potential sample dropout rate of 20%, a minimum of 15 participants were recruited for this study. Accordingly, 18 highly trained male sprinters were recruited and one withdrew due to an accidental ankle sprain sustained during daily living activities (unrelated to the experimental protocol). The final 17 participants [age (22.88 ± 1.13) years, height (178.46 ± 3.24) cm, mass (72.12 ± 3.74) kg, thigh circumference (56.67 ± 1.77) cm, mean applied BFR inflation pressure (271.26 ± 28.85) mmHg, years of training (4.31 ± 1.29), and 1RM hip thrust (HT) (196.41 ± 35.28) kg, 1RM barbell half squat (HS) (150.19 ± 19.92) kg] completed all the tests. Participants were excluded if they met any of the following criteria: (1) a history of any neuromuscular or musculoskeletal injuries (e.g., lower limb fractures, ligament tears) within the past six months that could impede exercise performance, regardless of whether the injury was sports-related; (2) any diagnosed cardiovascular, metabolic, or respiratory disorders that serve as contraindications to high-intensity resistance training; or (3) current smoking habits.

In addition, participants were recruited on a voluntary basis, and prior to enrollment, each individual received a detailed explanation of the study objectives, procedures, and potential risks involved. Only after fully understanding this information did participants provide written informed consent, thereby confirming their willingness to take part in the study. All participants were instructed to: 1) avoid high-load physical activities, consumption of caffeine- or alcohol-containing beverages 24 hours prior to all measurements and ensure at least 8 hours of sleep [30]; 2) maintain similar training session during this study; 3) wear standard athletic clothing and footwear; 4) refrain from engaging in high-load training activities during the washout period.

The study was conducted in accordance with the Declaration of Helsinki. Approval for this study was obtained from the Ethics Committee of Beijing Sport University (2023215H) and informed consent was obtained from all participants. The recruitment period for this study was from May 1, 2023 to June 1, 2023.

### Procedures

A randomized crossover controlled trial design was used in this study. Before the pre-test session, participants completed a familiarization phase to minimize potential learning effects. During the initial visit, participants received comprehensive instruction on the study protocols and testing procedures. Following this induction, thigh circumference was measured at the midpoint of the thigh to determine individualized BFR pressures. Participants then performed their first one-repetition maximum (1RM) test (HS or HT). To ensure valid strength assessment without fatigue interference, the 1RM tests for the HS and HT were conducted on separate days, separated by a 72-hour recovery interval. The execution order of these two strength tests was determined randomly for each participant using a lottery method. These baseline strength values were used to calculate the specific training loads (30% and 90% 1RM) for the subsequent intervention.

During the pre-test session, participants first performed an 8–10 minutes standardized warm-up, including jogging, glute activation exercises, dynamic stretching, and marching movement integration [31]. They then rested for 3 minutes before completing the pre-CA assessments. To prevent fatigue interference, measurements were conducted in the following specific order: first, pennation angle assessment and muscle–tendon stiffness testing, followed by the 20 m sprint test. Additionally, all assessments for each participant were scheduled within a consistent time window (maximum variation ± 1 hour) to minimize the influence of circadian rhythms.

**Fig 1:**
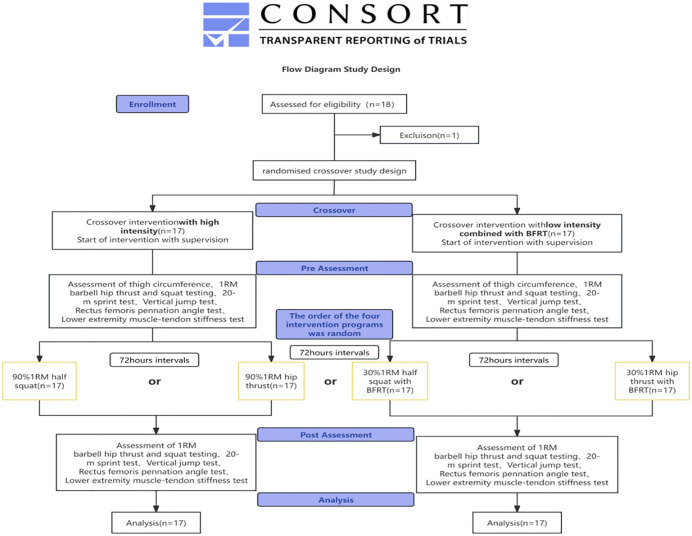
Study flow diagram. The diagram (The CONSORT: Consolidated Standards of Reporting Trials) includes detailed information on the interventions received.

In the formal assessment session, participants completed four intervention conditions in a randomized order: (i) 90% 1RM barbell HT, (ii) 90% 1RM barbell HS, (iii) 30% 1RM HT with BFR, and (iv) 30% 1RM HS with BFR. These two exercises were specifically selected to represent distinct force vector profiles relevant to sprinting mechanics: the hip thrust emphasizes anteroposterior force production (hip extension), whereas the half-squat primarily targets vertical force production. Integrating both exercises allows for a comprehensive comparison of how different loading vectors, combined with BFR, influence explosive performance. Each participant completed a total of 12 separate intervention trials (4 conditions × 3 testing parameters) in a randomized order. To ensure that every outcome measure (sprint performance, muscle stiffness, or pennation angle) was assessed precisely at the optimal potentiation window of 5 minutes post-intervention [32,33], only one variable was tested per trial. This rigorous design eliminated the timing delays inherent in sequential testing. A 72-hour washout period was strictly maintained between consecutive trials to prevent carry-over effects and fatigue [34,35]. A schematic of the experimental protocol is shown in Fig 1.

These interventions were implemented using two resistance exercise protocols, differing in load and the application of BFR. The low-load protocol incorporated BFR and consisted of 30% 1RM hip thrust (HT) or half-squat (HS) performed for 15 repetitions per set across three sets, with 30 s inter-set rest, following the procedures of Abe et al.(2005) [36] and Patterson et al.(2019) [37]. The high-load protocol involved 90% 1RM HT or HS for one set with three repetitions [38].

To minimize the potential for interaction between interventions, participants were explicitly instructed to refrain from engaging in high-load training activities during the washout period and to ensure their readiness for each training session. All testing was conducted under stable environmental conditions, with room temperature ranging from 23.2°C to 26.3°C and relative humidity from 67% to 85%.

#### Selection of blood flow restriction equipment model, operation method and training pressure.

The blood flow restriction equipment used was the B STRONG pressurized training belt (B STRONG, Utah, USA), featuring an adjustable design and employing a distributed airbag pressure filling method. For the intervention, a 5-cm wide tourniquet cuff was securely positioned at the most proximal portion of the thigh (groin crease). The same applied pressure does not produce an equivalent degree of blood flow restriction in all individuals, as the extent of restriction depends on cuff width [39] and individual characteristics such as limb circumference [40]. Therefore, using an absolute or uniform pressure for blood flow restriction training may be inappropriate. Some studies have proposed BFR training pressures based on directly measured thigh arterial occlusion pressure and thigh circumference [40], and subsequent research has confirmed beneficial training effects using this approach. Accordingly, this pressure selection method was adopted in the present study.

Occlusion pressure was prescribed based on thigh circumference as follows: 200 mmHg for < 45–50 cm, 250 mmHg for 51–55 cm, 300 mmHg for 56–59 cm, and 350 mmHg for > 60 cm. The mean BFR training pressure applied across participants was 271.26 ± 28.85 mmHg. Given this relatively high occlusion pressure and the multiple visits required, an intermittent BFR protocol was employed to optimize participant tolerance. The cuffs were inflated during exercise sets but were deflated (maintained at ~30 mmHg) during the inter-set rest intervals [37].

#### Barbell half squat and barbell hip thrust 1RM test.

For the Smith half squat 1RM test, participants positioned their feet slightly wider than shoulder width, rotated their toes outward, and descended until their thighs were parallel to the floor [41]. A clinometer was used to strictly monitor the knee angle and standardize the squat depth. A certified strength and conditioning specialist (NSCA-CSCS) monitored the technique and provided verbal cues to ensure safety.

During the hip thrust test, a soft cushion was positioned at the participant’s anterior superior iliac spine, and the upper back was placed on a training bench. The feet were positioned slightly wider than shoulder width, with toes externally rotated. The movement involved lowering the barbell until it touched the ground, while maintaining a neutral spine and pelvis position on the ascent [30].

Both 1RM assessments followed a standardized progression aligned with National Strength and Conditioning Association (NSCA) guidelines. Participants initially attempted a weight they could easily lift for 5–10 repetitions, followed by a 2-minute rest. Subsequent attempts increased the weight by 10–20% each time, with a 2–4-minute rest between sets. The subject’s 1RM was determined within 3–5 attempts.

#### 20m sprint test.

Participants conducted the 20m sprint test on the track using portable Smart Speed timing gates (Smart Speed Pro, Fusion Sport, Australia), positioned at 0m, 10m, and 20m, and configured in running application mode. To prevent interference with the timing system, athletes assumed a three-point pre-sprint position 30 cm behind the starting line, ensuring their bodies did not cross the starting infrared beam [30]. The test was conducted three times with 2–3 minute intervals between attempts, and the best performance was selected for statistical analysis.

#### Rectus femoris pennation angle test.

The pennation angle of the rectus femoris muscle in athletes was measured using a GE-LOGIQ-E9 color ultrasound diagnostic device (GE LOGIQ-E9, Wauwatosa, WI, USA). Following the guidelines of the American Institute of Ultrasound Medicine, athletes were instructed to wear shorts, relax their legs, and lie in a supine position with the femur in a neutral orientation. To ensure consistency, all ultrasound images were acquired by the same experienced investigator. Subsequently, image analysis was performed by a separate investigator who was blinded to the specific intervention condition associated with each image to minimize potential bias. Ultrasound gel was applied to a 12-MHz linear probe, which was positioned along the long axis of the anterior thigh. Measurements were obtained from the muscle belly of the rectus femoris on the dominant leg, at two-thirds of the distance between the anterior superior iliac spine and the superior border of the patella [42]. The probe was placed as close as possible to the muscle belly, and the image depth was set to 5 cm to optimize visualization. Test timing was consistent with the protocol described earlier.

**Fig 2:**
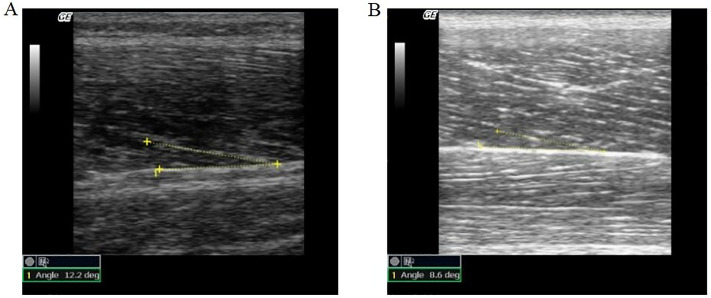
The pennation angle change test picture. Panel (A) shows the initial measurement of the pennation angle (12.2°), while panel (B) shows the pennation angle after the intervention (8.6°).

A custom-made ultrasound probe fixation device ensured the probe was aligned parallel to the muscle fibers’ direction, and skin markers were drawn on the thigh to ensure reproducibility across assessments. The result example in the change of pennation angle is shown as Fig 2.

#### Lower extremity muscle-tendon stiffness test.

Higher stiffness facilitates the stretch-shortening cycle (SSC) and higher velocity explosive movements. Therefore, testing the stiffness of lower limb muscles and tendons is crucial for assessing the effectiveness of preparatory activities. The Myoton PRO (Myoton AS, Tallinn, Estonia) portable diagnostic equipment for muscle function utilizes parameters and measurements referenced from the study conducted by Papla et al.(2023) [17]. The device’s accelerometer was set to 3200 Hz, and the average value was calculated from 3 consecutive measurements, each monitored with a 3% error margin. During testing, the Myoton PRO probe was perpendicular to the surface of each measurement point (duration: 15 ms, force: 0.58 N) [14]. This study primarily focused on testing the rectus femoris, biceps femoris, medial gastrocnemius, lateral gastrocnemius, and tibialis anterior muscles. The specific measurement sites were as follows: rectus femoris (50% of the distance between the anterior superior iliac spine and the superior border of the patella), biceps femoris (midpoint between the ischial tuberosity and the lateral tibial epicondyle), medial gastrocnemius (at the point of maximal bulge), (one-third of the distance from the fibular head to the heel), tibialis anterior (one-third of the distance between the fibular head and the medial malleolus), and the Achilles tendon (5 cm above the calcaneus). The testers recorded the test position with a marker that is not easy to scrub to ensure that the test position is consistent each time. participants were instructed to fully relax their muscles during the measurement process to standardize muscle activity.

### Statistical analysis

All data were analyzed using IBM SPSS Statistics for Macintosh, Version 22.0 (IBM Corp., Armonk, N.Y., USA) and were presented as mean ± standard deviation (± SD). Normality of data distribution was verified using Shapiro–Wilk tests. Mauchly’s test of sphericity was used to test the assumption of sphericity. If the sphericity assumption was violated (P < 0.05), the Greenhouse–Geisser correction was applied; otherwise (P > 0.05), the results based on the assumption of sphericity were used. The main effects of group (intervention group) and time (testing time point), as well as the group × time interaction effect, were examined. When a significant interaction was found, simple effects were analyzed using Bonferroni‑corrected tests. Statistical comparisons were conducted both within groups (post‑test vs. baseline) and between groups (post‑intervention).The magnitude of mean differences was expressed with standardized effect size (ES). Thresholds for qualitative descriptors of Hedges g were interpreted as ≤ 0.20 “small”, 0.21–0.79 “medium”, and > 0.80 as “large” [15]. Statistical significance was set at *P* < 0.05.

## Results

### 20m sprint

**Fig 3:**
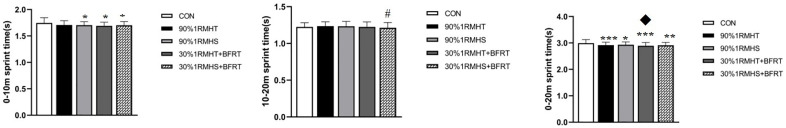
Effect of different exercise interventions on the sprint time of 20m segments. The figure illustrates the sprint times for the 0-10m, 10-20m, and 0-20m segments, highlighting the effects of different exercise interventions on sprint performance.

Repeated ANOVA showed statistically significant differences among interventions for both 10–20 m sprint (*F* = 4.632, *P* < 0.05, η^2^ = 0.22) and 0–20 m sprint performance (*F* = 12.58, *P* < 0.05, η^2^ = 0.44). As shown in Fig 3, the post-hoc analysis indicated that after 30% 1RM HT + BFRT, the 0–20 m sprint ability was significantly improved compared to 90% 1RM HS (2.89 ± 0.12 s vs. 2.93 ± 0.10 s, *P* < 0.05, *ES* = 0.36, Δ% = 1.3%). Additionally, after 30% 1RM HS + BFRT, the 10–20 m sprint ability was significantly improved compared to both 90% 1RM HT (1.21 ± 0.06 s vs. 1.24 ± 0.05 s, *P* < 0.05, *ES* = 0.54, Δ% = 2.5%) and 90% 1RM HS (1.21 ± 0.06 s vs. 1.23 ± 0.06 s, *P* < 0.05, *ES* = 0.34, Δ% = 1.7%).

### Rectus femoris pennation angle

**Fig 4:**
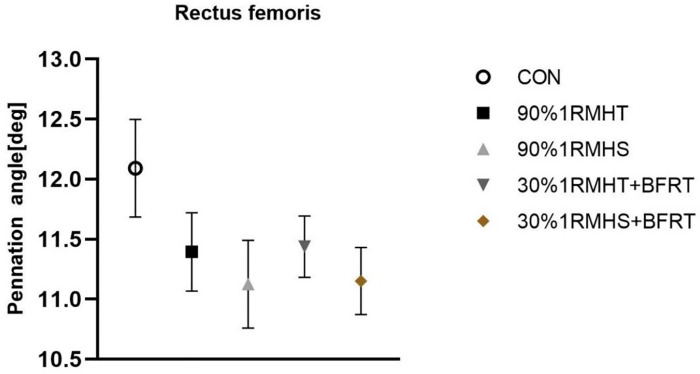
Effect of different exercise interventions on the pennation angle of rectus femoris muscle. The figure shows the pennation angle (degrees) for CON, 90% 1RM HT, 90% 1RM HS, 30% 1RM HT, 30% 1RM HS, and 30% 1RM HS + BFR. Note: the up and down directions of the triangles are used solely as symbols to distinguish between different experimental groups. The orientation of the triangles does not represent the direction of change in the measured variable.

As demonstrated in Fig 4, the repeated-measures ANOVA revealed a significant main effect of group on the change in rectus femoris pennation angle (*F* = 13.91, *P* < 0.0001, η² = 0.47).Post-hoc analysis indicated that after the 30% 1RM HS + BFRT, the rectus femoris pennation angle significantly decreased compared to baseline (11.15 ± 0.27° vs. 11.44 ± 0.25°, *P* = 0.043, *ES* = 1.11, Δ% = 2.6%). A similar decrease was observed after the 90% 1RM HS (11.13 ± 0.36° vs. 11.44 ± 0.25°, *P* = 0.039, *ES* = 1.00, Δ% = 2.8%), when compared to the 30% 1RM HT + BFRT group. No significant differences were found between the other interventions (*P* > 0.05).

### Lower limb muscle-tendon stiffness

**Fig 5:**
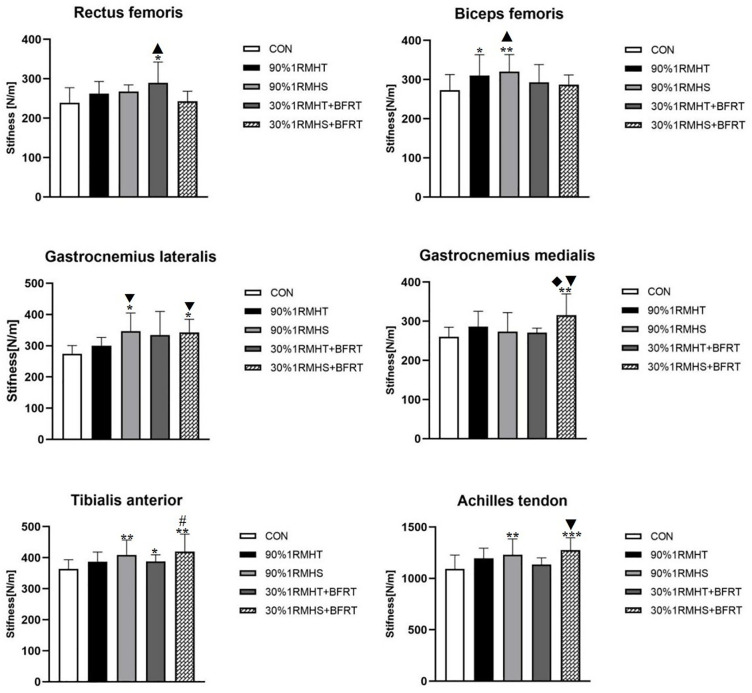
Effect of different exercise interventions on lower limb muscle-tendon stiffness. The figure shows stiffness measurements (N·m) for the Rectus femoris, Biceps femoris, Gastrocnemius lateralis, Gastrocnemius medialis, Tibialis anterior, and Achilles tendon following different interventions: CON, 90% 1RM HT, 90% 1RM HS, 30% 1RM HT, 30% 1RM HS, and 30% 1RM HS + BFR.

As illustrated in Fig 5, repeated-measures ANOVA revealed significant difference in the effects of different interventions on rectus femoris muscle stiffness (*F* = 3.43, *P* = 0.01,η^2^ = 0.18), biceps femoris muscle stiffness (*F* = 5.85, *P* = 0.02, η^2^ = 0.27), lateral gastrocnemius head stiffness (F = 4.038, *P* = 0.007, η^2^ = 0.20), medial gastrocnemius head stiffness (*F* = 5.668, *P* = 0.004, η^2^ = 0.26), tibialis anterior muscle stiffness (*F* = 13.37, *P* = 0.04, η^2^ = 0.46), and Achilles tendon stiffness (*F* = 6.013, *P* = 0.003, η^2^ = 0.27).

Post-hoc comparisons further indicated that after the 30% 1RM HT + BFRT, rectus femoris muscle stiffness was significantly higher compared to the 30% 1RM HS + BFRT (289.80 ± 52.74 N/m vs. 242.70 ± 25.80 N/m, *P* = 0.04, *ES* = 1.13, Δ% = 19.4%). Additionally, following the 90% 1RM HS, biceps femoris stiffness was significantly higher compared to 30% 1RM HS + BFRT (320.10 ± 43.91 N/m vs. 287.10 ± 24.93 N/m, *P* = 0.03, *ES* = 0.92, Δ% = 11.5%).

Regarding the medial gastrocnemius head stiffness, after the 30% 1RM HS + BFRT, significant increases were observed compared to both 90% 1RM HS (315.50 ± 54.51 N/m vs. 273.60 ± 48.39 N/m, *P* = 0.01, *ES* = 0.81, Δ% = 15.3%) and 30% 1RM HT + BFRT (315.50 ± 54.51 N/m vs. 271.10 ± 11.31 N/m, *P* = 0.006, *ES* = 1.13, Δ% = 16.4%). In addition, after the 30% 1RM HS + BFRT, tibialis anterior muscle stiffness was significantly higher compared to 90% 1RM HT (387.0 ± 31.15 N/m vs. 419.80 ± 56.10 N/m, *P* = 0.02, *ES* = 0.72, Δ% = 8.5%).

Furthermore, Achilles tendon stiffness was significantly greater after the 30% 1RM HS + BFRT compared to 30% 1RM HT + BFRT (1275.05 ± 120.30 N/m vs. 1134.16 ± 66.36 N/m, *P* = 0.012, *ES* = 1.45, Δ% = 12.4%).

Regarding changes from baseline, significant increases were noted in several stiffness measures. After the 30% 1RM HT + BFRT, rectus femoris muscle stiffness was significantly higher compared to baseline (289.80 ± 52.74 N/m vs. 239.30 ± 39.03 N/m, *P* = 0.026, *ES* = 1.09, Δ% = 10.9%). Similarly, biceps femoris stiffness was significantly greater after both 90% 1RM HT (310.0 ± 53.41 N/m vs. 273.30 ± 39.57 N/m, *P* = 0.02, *ES* = 0.78, Δ% = 13.4%) and 90% 1RM HS (320.10 ± 43.91 N/m vs. 273.30 ± 39.57 N/m, *P* = 0.001, *ES* = 1.12, Δ% = 17.1%).

For lateral gastrocnemius head stiffness, significant increases were observed after the 90% 1RM HS (346.90 ± 57.91 N/m vs. 274.10 ± 26.61 N/m, *P* = 0.02, *ES* = 1.61, Δ% = 26.5%) and 30% 1RM HS + BFRT (342.90 ± 41.70 N/m vs. 274.10 ± 26.61 N/m, *P* = 0.03, *ES* = 1.97, Δ% = 25.1%) compared to baseline. Additionally, lateral gastrocnemius stiffness was significantly greater after 90% 1RM HS compared to 90% 1RM HT (346.90 ± 57.91 N/m vs. 299.70 ± 27.16 N/m, *P* = 0.03, *ES* = 1.04, Δ% = 15.7%), and after 30% 1RM HS + BFRT compared to 90% 1RM HT (342.90 ± 41.70 N/m vs. 299.70 ± 27.16 N/m, *P* = 0.05, *ES* = 1.23, Δ% = 14.4%).

Moreover, after the 30% 1RM HS + BFRT intervention, medial gastrocnemius head stiffness was significantly higher compared to baseline (315.50 ± 54.51 N/m vs. 260.20 ± 2.60 N/m, *P* = 0.0003, *ES* = 1.43, Δ% = 21.3%).

Finally, tibialis anterior muscle stiffness was significantly increased after the 30% 1RM HS + BFRT compared to baseline (419.80 ± 56.10 N/m vs. 363.70 ± 29.65 N/m, *P* = 0.003, *ES* = 1.25, Δ% = 15.4%), and significantly improved after 30% 1RM HT + BFRT compared to baseline (388.0 ± 21.26 N/m vs. 419.80 ± 56.10 N/m, *P* = 0.027, *ES* = 0.75, Δ% = 8.2%).

## Discussion

The aim of this study was to analyze the acute effect of 30% 1RM with BFR vs. 90% 1RM without BFR and hip thrust vs. barbell half squat on sprint ability, pennation angle, and lower extremity muscle-tendon stiffness in sprinters. The influence of post activation potentiation on lower limb muscle tendon stiffness and muscle pennation angle in male sprinters with systematic training experience remains unclear, particularly in terms of the changes in muscle-tendon stiffness and pennation angle following PAPE induced by different interventions. Understanding these effects may provide a new perspective on how PAPE enhances subsequent athletic performance, offering significant practical value and interest.

### Effects of sprint ability

The ability to sprint 20m is a critical determinant of sprint performance. Based on significant changes in gait characteristics during the 0-20m sprinting process, this ability can be further divided into start-up speed (0-10m) and acceleration (0-20m). The results indicated that after 30% 1RM HT + BFR, the 0-20m sprint ability was significantly improved compared to the 90% 1RM HS. These findings suggest that combining low-load hip thrust resistance exercise with blood flow restriction can acutely enhance speed performance (both start-up speed and acceleration), with a notably stronger acute effect on acceleration capacity compared to high-load resistance training. The study by Eserhaut et al. (2025) [43]reported that both low-load blood flow restricted resistance exercise and high-load resistance exercise significantly elevated the potent β2 adrenergic receptor (β2AR) agonist EPI (IP: 1.29 ± 0.44 and 1.35 ± 0.60 nmol·L^−1^, respectively) as well as the androgenic steroid T (+5 min: 27.4 ± 12.9 and 29.0 ± 14.3 nmol·L^−1^, respectively) in well‑trained males. In the present study, low‑load hip thrust resistance exercise combined with blood flow restriction demonstrated a greater acute enhancement of both starting speed and acceleration performance. This phenomenon may be attributed to two primary factors. First, the significant improvement in acceleration performance induced by low‑intensity resistance exercise with blood flow restriction may be related to acute increases in muscular strength. During blood flow restriction training, insufficient oxygen supply to slow‑twitch fibers leads to earlier recruitment of fast‑twitch fibers [44]. Second, the barbell hip thrust markedly increases the horizontal force vector during sprinting [45], thereby better developing force production in the anteroposterior direction; importantly, force output in the horizontal direction has been shown to be more closely related to sprint performance than force in the vertical direction [46].

This study further confirmed that 10–20 m sprint performance following a 30% 1RM hip thrust with BFR was significantly improved compared to the 90% 1RM hip thrust condition. This finding contrasts with those reported by Atalağ et al. (2020) [47] and Dello et al. (2018) [48]. The discrepancy may be attributed to differences in participant sex, training background, recovery intervals, and the intensity of the potentiating stimulus.In the present study, data from high-level male sprinters were collected 5 minutes after a single set of three repetitions at 90% 1RM barbell hip thrust. In contrast, Atalağ et al. (2020) examined resistance-trained university students (8 males, 9 females) using an identical loading protocol (one set of three repetitions at 90% 1RM) but assessed performance 8 minutes post-intervention. Dello et al. (2018) employed a different protocol involving 18 male soccer players, who performed three sets of six repetitions at 85% 1RM. Performance was tested at 15 seconds, 4 minutes, and 8 minutes after the intervention-a design in which successive measurements could have interfered with each other. These comparisons underscore the complexity and ongoing debate surrounding the influence of load intensity, post-activation assessment timing, and athlete training status on the PAPE effect for sprint performance.

The results of the study demonstrated that combining low-load weight-bearing half-squats with blood flow restriction resulted in a significant augmentation effect on sprint performance. These findings align with a study by Chen et al. (2021) [49], which investigated the effects of running exercise (50% heart rate reserve, 2 minutes × 5 sets, 1-minute rest interval) combined with BFR (occlusion pressure: 1.3 × resting systolic blood pressure) on sprint performance in 12 male sprinters. Sprint performance was assessed after a 5-minute recovery from the experimental intervention, the results showed an improving trend in both 10-meter and 10–30-meter sprint performance, with the improvements not reaching statistical significance but exhibiting a moderate effect size (ES > 0.50). Short-distance acceleration capacity involves rapidly accelerating from a stationary or slow state to maximal velocity, requiring explosive strength and maximal strength capacity. Low-intensity resistance exercise combined with blood flow restriction training can enhance neural activation and increase recruitment of motor units of type II muscle fibers during exercise [50]. Furthermore, after BFR training, an increase in anabolic hormone secretion and a significant elevation of IGF-1 concentration have been observed [51], both of which are associated with improvements in strength and power. In pre-competition warm-ups or activation routines before speed training, when the goal is to enhance short-distance acceleration ability, 30% HT + BFR holds greater practical potential.

### Effect of muscle pennation angle

In anatomy, the angle between the tendon and the muscle fibers is commonly referred to as the “pennation angle”. This angle indicates the orientation of the muscle fibers in relation to the tendon [52]. Upon skeletal muscle contraction, the total force transmitted from individual muscle fibers to the tendons and bones increases as the pennation angle decreases. Folland et al. (2007) [53] demonstrated that the size of the pennation angle affects the efficiency of force transmission from muscle to tendon and bone. Additionally, the size of pennation angle also influences the relationship between the force and velocity curves, with a smaller angle being more favorable for force transmission and subsequently enhancing power output. Consequently, a decrease in pennation angle results in increased muscle contraction force. The results of the current study demonstrated that both the 90% 1RM HS and 30% 1RM HS + BFR exercise interventions significantly reduced the pennation angle of the rectus femoris muscle. Similarly, Mahlfeld et al.(2004) [5] reported that the pennation angle was slightly, though not significantly, reduced immediately following MVIC exercise. However, 3–6 min later, a significant reduction in pennation angle was observed. This change would only be equivalent to a 0.9% increase in force transmission to the tendons, but it is possible that this

effect may contribute to PAP [1]. Kubo et al. [54]suggest that conditioned contraction can increase adherence to connecting tissues or tendons, which may offset the increase in force transmission caused by any reduction in pinnate angle. It triggers changes in neuromuscular mechanisms, enhances muscle activation and increases nerve excitability, which jointly explains the reasons for the increase in explosive performance. Consequently, when the primary objective is to modulate the pennation angle of the rectus femoris muscle or to prepare for specific sprint phases, 90% 1RM HS and the 30% 1RM HS + BFR may be more appropriate.

### Effects of lower limb muscle-tendon stiffness

The Myoton PRO Digital Muscle Function Testing System is a portable device that offers a novel method for assessing lower limb muscle-tendon stiffness in high-level athletes. Numerous studies have demonstrated a positive correlation between tendon stiffness and jumping performance, indicating that greater stiffness is advantageous for activities involving stretch-shortening cycles (SSC) and sports that require higher speeds [11,55]. Participants with greater stiffness have shown enhanced locomotor performance during SSC activities.

Biel et al. conducted a study aimed at assessing the acute effects of unilateral and bilateral conditioning activities on vastus lateralis stiffness, countermovement jump parameters, and 10 m sprint performance. The results showed that increases in vastus lateralis stiffness were observed across conditions or time points, but none reached statistical significance [15]. In contrast, Bojsen‑Müller et al. (2005) [16] reported that higher stiffness not only facilitates the storage and release of elastic energy and the rate of force development but is also crucial for muscle force generation. The findings of the present study indicate that following 30% 1RM hip thrust combined with BFR, rectus femoris stiffness was significantly enhanced. Furthermore, biceps femoris stiffness was significantly greater following 90% 1RM hip thrust, and lateral gastrocnemius head stiffness was significantly greater after both 90% 1RM hip thrust and 30% 1RM HS + BFR. Additionally, medial gastrocnemius head stiffness was significantly increased after 30% 1RM HS + BFR, while tibialis anterior muscle stiffness and Achilles tendon stiffness were significantly increased after 30% 1RM HS + BFR. Byrne et al.(2020) [56] demonstrated that drop jump exercise completion resulted in shorter countermovement jump (CMJ) touchdown times, increased reactive strength index (RSI) and enhanced muscle-tendon unit stiffness properties. This suggests that SSC performance was enhanced during CMJ, leading to significant increases in CMJ height. Muscle -tendon stiffness can not only improve the transmission efficiency of force, which is consistent with the fact that in the process of skeletal muscle contraction, the more active contraction reduces the length of muscle fibers, the smaller the pinnate angle formed, the more conducive to improving the transmission efficiency of force, and there seems to be a superimposed effect of superimposing to improve explosive force performance. Thus, when the focus is more on lower limb stiffness modulation or preparation for specific sprint phases, 90% 1RM HS and 30% 1RM HS + BFR might be more suitable.

From a practical standpoint, the 5‑minute recovery window observed in this study provides a useful reference for coaches and athletes. When incorporating low‑load BFR conditioning activities into pre‑competition warm‑ups or speed training sessions, a 5‑minute interval may allow sufficient recovery from fatigue while preserving the potentiation effect. Applying the stimulus too early could result in residual fatigue, whereas waiting too long may lead to decay of the potentiation benefit. Therefore, to optimize acute sprint performance, athletes are advised to complete the potentiation‑inducing conditioning activity approximately 5 minutes prior to the actual sprint or competition start.

## Limitations of the study

The present study has limitations concerning both sample generalizability and experimental control. Given that the investigation was conducted exclusively on male university sprinters, caution is warranted when extrapolating these findings to female athletes or elite-level populations. Methodologically, it is important to acknowledge that our protocol prioritized the comparison between specific high-load and BFR-combined training strategies. This focus resulted in the absence of a work-matched low-load control (without BFR) or a passive control group, which limits the ability to strictly isolate the independent physiological contributions of blood flow restriction versus the mechanical stimulus of exercise itself. Additionally, despite our efforts to counterbalance the order of experimental conditions, the repeated-measures design may still introduce potential order or fatigue effects across trials, which could have influenced the performance outcomes.

Regarding the physiological assessment, this study primarily evaluated sprinting time, muscle pennation angle, and lower limb muscle-tendon stiffness, but did not analyze physiological indices such as EMG signals, electroencephalography, or blood lactate concentration. Consequently, the explanatory power for determining the precise mechanistic drivers of the acute changes is constrained. Furthermore, related to the measurement of muscle-tendon stiffness, the Myoton PRO system is restricted to assessing superficial tissues under static conditions. This limitation not only precludes the quantification of deep-seated muscles but also implies that the obtained values reflect a resting mechanical state, which may not fully capture the dynamic modulation of stiffness occurring during high-velocity sprinting.

## Conclusions

This study demonstrates that in male sprinters, low‑load blood flow restriction training (BFR) produces acute improvements in sprint performance, pennation angle, and muscle‑tendon stiffness that are comparable to or slightly greater than those induced by high‑load training. Specifically, low‑load BFR combined with barbell hip thrust enhances sprint ability, while low‑load BFR with barbell half‑squat reduces rectus femoris pennation angle and increases lower‑limb stiffness. The concurrent reduction in pennation angle and increase in stiffness may jointly explain the observed post‑activation performance enhancement and explosive power gains.
